# A Phase Ib Trial of Copanlisib in Combination with Venetoclax in Patients with Relapsed or Refractory B-Cell Non-Hodgkin Lymphoma—SAKK 66/18 Trial

**DOI:** 10.3390/cancers18111764

**Published:** 2026-05-28

**Authors:** Maria Cristina Pirosa, Dagmar Hess, Fatime Krasniqi, Urban Novak, Nicolas Mach, Thorsten Zenz, Lisa Holer, Luciano Cascione, Eleonora Cannas, Andrea Rinaldi, Sämi Schär, Emanuele Zucca, Francesco Bertoni, Anastasios Stathis

**Affiliations:** 1 Institute of Oncology Research, Faculty of Biomedical Sciences, Università della Svizzera Italiana, 6500 Bellinzona, Switzerlandemanuele.zucca@ior.usi.ch (E.Z.);; 2 Oncology Institute of Southern Switzerland, Ente Ospedaliero Cantonale, 6500 Bellinzona, Switzerland; 3 Faculty of Biomedical Sciences, Università della Svizzera Italiana, 6900 Lugano, Switzerland; 4 Kantonsspital St. Gallen, 9007 St. Gallen, Switzerland; 5 Universitätsspital Basel, 4031 Basel, Switzerland; 6 Department of Medical Oncology, Inselspital, Bern University Hospital, University of Bern, 3010 Bern, Switzerland; 7 Hôpitaux Universitaires de Genève, 1205 Genève, Switzerland; nicolas.mach@hcuge.ch; 8 Universitätsspital Zürich, 8091 Zürich, Switzerland; 9 Competence Center of SAKK, 3008 Bern, Switzerland; 10 SIB Swiss Institute of Bioinformatics, 1015 Lausanne, Switzerland

**Keywords:** early-phase trial, B-cell lymphoma, PI3K inhibitor, BCL2 inhibitor

## Abstract

**Background**: This phase I trial aimed to assess the safety and preliminary activity of the combination of copanlisib with venetoclax in previously treated patients with B-cell non-Hodgkin lymphoma, excluding mantle-cell lymphoma patients. **Methods**: Intravenous copanlisib on days 1, 8, and 15 and oral venetoclax once daily (starting on day 2 of cycle 1, continuously) were administered in 28-day cycles up to a maximum of 12 cycles. Starting doses were 600 mg of venetoclax and 60 mg of copanlisib. **Results**: A total of seven patients were enrolled. The first two presented dose-limiting toxicities, five additional patients were treated at a lower dose level (45 mg of copanlisib and 400 mg of venetoclax), with no dose-limiting toxicities observed among three evaluable patients. The most frequent treatment-related adverse event of grade 3 or higher was neutropenia (*n* = 3), followed by thrombocytopenia (*n* = 2) and hypertension (*n* = 2). Four treatment-related serious adverse events occurred, including grade 3 febrile neutropenia in one patient and respiratory infections in three patients. Two patients had complete responses, and two had partial responses. Transcriptomic analysis of lymphoma samples showed enrichment of genes coding for proteins involved in B-cell receptor signaling in responding patients. The study was terminated prematurely due to a decision by the pharmaceutical company. **Conclusions**: Due to the limited sample size, no definite conclusions regarding safety or activity of the combination can be drawn. The two drugs could not be combined at their respective single-agent doses. Responses were observed in heavily pretreated patients. Transcriptomic analyses suggested a possible association between response and B-cell receptor signaling, which warrants further investigation.

## Simple Summary

Patients with B-cell non-Hodgkin lymphoma that has returned or no longer responds to treatment still have limited therapeutic options. This study aimed to explore whether the combination of two targeted drugs—copanlisib and venetoclax—could be safe and effective in these patients. Both drugs have shown activity when used alone, and laboratory studies have suggested they might work better together. However, in this small clinical trial, the combination caused relevant side effects that required dose reductions, and the study was stopped early. Despite these challenges, some patients experienced tumor shrinkage, including complete responses. Importantly, analysis of tumor samples suggested that patients whose lymphoma relied on specific signaling pathways were more likely to benefit. These findings highlight both the potential and the limitations of combining targeted therapies and suggest that selecting patients based on tumor biology may help improve future treatment strategies.

## 1. Introduction

Non-Hodgkin B-cell lymphomas (B-NHLs) include several subtypes; among aggressive lymphomas, diffuse large B-cell lymphoma (DLBCL) is the most frequent, while follicular lymphoma (FL) is the most frequent indolent subtype [1,2,3]. In recent years, many new active therapies have been developed for patients with relapsed/refractory DLBCL, including new monoclonal antibodies, bispecific antibodies, and chimeric antigen receptor (CAR) T cells [4,5,6,7]. However, many patients are still not cured. Among patients with indolent lymphomas, those with early relapse following first-line therapy have an unfavorable prognosis. New treatment approaches, including the use of novel monoclonal antibodies, antibody–drug conjugates, bispecific antibodies, small molecules, and combinations of new drugs, are being actively explored.

Targeting of BCL2 protein and PI3K has been very effective across different lymphoma subtypes, although, more recently, concerns about adverse events have emerged, particularly with regard to PI3K inhibitors, leading to the withdrawal of drugs previously approved for indolent lymphomas [8,9]. Copanlisib, a pan-PI3K inhibitor with dominant activity against PI3Kα and PI3Kδ, has been approved by various regulatory agencies for adult patients with relapsed FL who have received at least two prior systemic therapies [10]. However, in November 2023, given the results of a phase III study that did not show sufficient improvement in progression-free survival (PFS) when copanlisib was combined with chemotherapy versus chemotherapy alone [9], the drug was voluntarily withdrawn from its indication in relapsed FL. Additionally, in February 2024, the EMA removed copanlisib’s orphan drug designation for relapsed/refractory (R/R) marginal-zone lymphoma (MZL) from the Community Register.

Overexpression of anti-apoptotic protein BCL-2, which impairs apoptosis, plays a key role in the pathogenesis of several NHL subtypes, and venetoclax has demonstrated clinical efficacy in chronic lymphocytic leukemia and mantle cell lymphoma [11]. Moreover, the PI3K pathway is critically involved in lymphoma biology, contributing to the proliferation and survival of malignant lymphoid cells, including FL and MZL [3].

The combination of copanlisib and venetoclax is therefore of interest because both agents have shown single-agent activity in B-cell NHL, and preclinical studies in B- and T-cell lymphoma models have demonstrated synergistic effects between the two compounds [12,13,14].

Here, we present the results of a phase I trial evaluating the safety and preliminary antitumor activity of copanlisib in combination with venetoclax in patients with B-cell NHLs. We also report the results of transcriptomic analysis on archived tumor samples from participating patients.

## 2. Materials and Methods

### 2.1. Study Design and Treatment

SAKK66/18 was an open-label, multicenter, dose-escalation phase Ib clinical trial of copanlisib in combination with venetoclax in patients with R/R B-cell NHLs (NCT03886649).

The primary objective was to determine the maximum tolerated dose (MTD) and the recommended phase II dose (RP2D) of copanlisib in combination with venetoclax in patients with R/R B-cell NHL. Secondary objectives included assessing safety and tolerability and evaluating preliminary antitumor activity in patients with R/R FL and MZL.

Patients with different NHL subtypes, excluding mantle cell lymphoma (MCL) and chronic lymphocytic leukemia (CLL)/small lymphocytic lymphoma (SLL), were eligible for the dose-escalation part of the study, which followed a standard 3 + 3 design.

During this phase, the decision to escalate to the next dose level was taken based on the number of reported Dose-Limiting Toxicities (DLTs) observed in the first cycle of trial therapy. In addition, all available safety data were considered before escalating to the next dose level. The estimated MTD was defined as the dose level below the one containing two or more DLTs. Upon identification of the MTD, 10 additional patients per cohort were to be enrolled in two cohorts: one with FL and one with MZL. The RP2D would be determined based on the combined safety data (dose escalation and expansion).

The initial study design consisted of treatment at dose level (DL) 1 with intravenous copanlisib 60 mg on days 1, 8, and 15 and oral venetoclax 600 mg QD continuously starting on day 2 of cycle 1 in cycles every 28 days for up to 12 cycles. Following the first 2 patients treated with the above-mentioned schedule, the study was amended to explore DL -1: copanlisib 45 mg on days 1, 8, and 15 and venetoclax 400 mg QD (cycles every 28 days).

DLTs were defined as treatment-related adverse events (AEs) occurring during the first cycle of treatment and fulfilling one of the following criteria: Grade 4 neutrophil count decreased (ANC < 0.5 × 10^9^/L for 7 or more consecutive days); Grade 3 febrile neutropenia (ANC < 1.0 × 10^9^/L and single temperature of >38.3 °C or a sustained temperature of ≥38.0 °C for >1 h); Grade 4 platelet count decreased (platelets < 25 × 10^9^/L); Grade 3 platelet count decreased (platelets < 50 × 10^9^/L) with clinically significant bleeding or requiring transfusion; Grade ≥ 3 laboratory abnormalities (including hyperglycemia) lasting for more than 7 days and assessed as clinically significant by the investigator; Grade ≥ 3 laboratory abnormalities consistent with tumor lysis syndrome (TLS) if associated with manifestations of clinical TLS; increased blood pressure that does not return to <150 mmHg (systolic blood pressure) and <90 mmHg (diastolic blood pressure) with or without the use of antihypertensive medication within 3 days of occurrence; trial treatment-related death; any trial treatment-related AE leading to ≥8 missed days (and doses) of venetoclax in cycle 1 or ≥ 2 missed doses of copanlisib in cycle 1 or a delay of >2 weeks in the start of cycle 2.

This study was performed in accordance with the principles of the Declaration of Helsinki and the International Conference on Harmonization Good Clinical Practice. The protocol was approved by the local ethics committee, and written informed consent was obtained from all participating patients.

### 2.2. Patients

Eligible patients were aged ≥18 years and had a histologically B-cell NHL diagnosis according to the WHO classification in the dose-escalation phase. In the expansion phase, only patients with FL or MZL could be enrolled. Additional inclusion criteria were relapsed or refractory disease after anti-CD20-based chemoimmunotherapy; absence of available effective standard treatment or refusal of such treatment by the patient; measurable disease according to Lugano classification [15]; ECOG performance status between 0 and 1; and adequate bone marrow, renal, and hepatic function.

Exclusion criteria included: diagnosis of CLL/SLL or mantle cell lymphoma (MCL) and other histologies requiring ramp-up of venetoclax; presence or history of central nervous system (CNS) disease (either CNS lymphoma or lymphomatous meningeosis) and primary CNS disease; prior treatment with venetoclax, copanlisib, or any other Bcl-2 inhibitors or PIK3 inhibitors; and treatment with any anti-lymphoma therapies within 21 days prior to registration (local radiation therapy for palliative treatment of symptoms was allowed).

### 2.3. Safety and Antitumor Activity Assessment

The screening had a duration of 28 days. During this period, the following tests were required: hematology (hemoglobin, ANC, and platelet count), blood chemistry (AP, AST, ALT, total bilirubin, serum creatinine, eGFR, Na, K, Ca, phosphate, uric acid, LDH, and fasting glucose), and HbA1c; IgG, IgA, and IgM; lipidic profile (triglycerides, LDL cholesterol, total cholesterol, lipase, and amylase) and coagulation parameters (PT/INR and aPTT); urine dipstick; PCR for CMV; serology for hepatitis B—HBsAg and total HBcAb—hepatitis C, and HIV; TSH, FT3, FT4; pregnancy test for women with child-bearing potential; radiology tumor assessment; bone marrow biopsy or aspirate only if clinically indicated; and cardiological assessment with ECG, echocardiogram, and physical examination. During treatment, laboratory analyses were performed on the same day as copanlisib administration; notably, on day 2, cycle 1, chemistry for TLS (creatinine, K, Ca, phosphate, and uric acid) was tested before the first dose of venetoclax, as well as 8 h and 24 h post dose.

Toxicity was graded using the National Cancer Institute Common Terminology Criteria for Adverse Events (NCI CTCAE) version 5.0.

Tumor assessment was performed at baseline and after 2, 6, 9, and 12 months by CT scan (with contrast unless contraindicated) or contrast-enhanced PET/CT.

Overall response (OR) was defined as the achievement of either complete response (CR) or partial response (PR), as assessed according to the Lugano classification [15]. Progression-free survival (PFS) was defined as the time from treatment initiation to the first documented disease progression (PD) or death from any cause—whichever occurred first.

### 2.4. Statistical Analyses

The MTD-determining analysis set was defined as all patients registered in the dose-escalation part, excluding patients who failed to satisfy major eligibility criteria; those who did not receive at least one dose of venetoclax and copanlisib, missed ≥2 doses of copanlisib or ≥8 days of venetoclax in the first cycle due to non-treatment-related reasons, and those who received strong or moderate CYP3A inhibitors or inducers during the DLT period. The full analysis set (FAS) was defined as all registered patients who received at least one dose of both trial treatments, excluding patients with major eligibility violations. The safety analysis set was defined as all patients who received any dose of trial treatment. In general, the summary statistics presented for quantitative variables were the median, minimum, and maximum values. The summary statistics presented for categorical data were the count and percentage of patients in each category. All analyses were performed using SAS 9.4 (SAS Institute Inc., Cary, NC, USA).

### 2.5. Transcriptome Profiling

RNA was extracted from two 5 μm formalin-fixed, paraffin-embedded (FFPE) sections or tissue rolls of representative tumor blocks at baseline using the Maxwell RSC RNA FFPE Kit (AS1440, Promega AG, Dübendorf, Switzerland), following the manufacturer’s protocol. RNA samples were then processed with the HTG EdgeSeq Oncology Biomarker Panel (HTG Molecular Diagnostics, Inc., Tucson, AZ, USA), following the manufacturer’s protocol. Sequencing was performed on a NextSeq 500 instrument (Illumina, San Diego, CA, USA) using the NextSeq 500/550 High Output Kit v2.5 (75 cycles). The RNA-Seq read quality was assessed with FastQC (v0.11.5) [16]. We removed low-quality reads and bases, as well as adaptor sequences, using Trimmomatic (v0.35) [17]. We aligned the trimmed, high-quality sequencing reads using STAR [18]. Samples were considered to be of good quality if we aligned more than 85% of the sequencing reads to the reference genome (HG38). The HTSeq-count software package (v. 0.13.5) [19] was used to quantify gene expression, using GENCODE v22 as the gene annotation. Sequencing data were imported in R (v. 4.4.2) and normalized with variance-stabilizing transformation (VST) from the DESeq2 package (v. 1.28.0) [20]. The differential gene expression for each comparison of interest was computed using limma based on TMM normalization and voom transformation [21]. Functional annotation was performed using the Gene Set Enrichment Analysis (GSEA) (v.4.3.3.) [22] on the *t*-test statistic pre-ranked list using the Hallmark and C2 Canonical Pathways gene sets from the Molecular Signatures Database (MSigDB), applying a threshold of FDR < 0.25 and *p* < 0.05. Expression data will be available in the National Center for Biotechnology Information Gene Expression Omnibus (http://www.ncbi.nlm.nih.gov/geo).

## 3. Results

### 3.1. Patient Characteristics

Between November 2019 and July 2020, seven patients were enrolled from six sites in Switzerland. The median age was 69 years (range 53–76). Lymphoma subtypes included FL (*n* = 4), DLBCL (*n* = 2), high-grade B-cell lymphoma with MYC and BCL2 and/or BCL6 rearrangements (HGBCL, *n* = 1). The median number of previous systemic treatments was 4 (range 2–6). Three patients had relapsed, and four had refractory disease. Baseline characteristics are reported in Table 1.

### 3.2. Treatment and Dose-Limiting Toxicities

All patients received at least one cycle of copanlisib and venetoclax. The median treatment duration was 7.1 (range 2–32) weeks. The median number of administered cycles was two (range 1–8). Reasons for treatment discontinuation included physician decision (*n* = 3, 42.9%), progressive disease (*n* = 3, 42.9%), patient’s refusal (*n* = 1, 14.3%), and unacceptable toxicity (*n* = 1, 14.3%).

The first two patients treated at DL1 presented DLTs (grade 3 febrile neutropenia in one patient; grade 3 maculo-papular rash and grade 3 upper respiratory infection in the other patient). Five patients were enrolled in DL -1, but two were not evaluable for DLTs (one withdrew consent during cycle 1, and one had major protocol violations during cycle 1). Among the three evaluable patients, no DLTs were reported (Table 2). However, even in the absence of DLTs, patients presented treatment-related AEs resulting in dose modifications or interruptions after cycle 1, and the plan was to introduce a further amendment in order to explore reduced doses and alternative schedules of the combination. However, the study was terminated prematurely due to a decision by the pharmaceutical company before the RP2D could be established, and no patients were enrolled in the planned dose-expansion cohorts.

### 3.3. Safety

All patients had at least one AE related to trial treatment of any grade. The most common treatment-related grade 1 AEs were nausea (four patients), dysgeusia (three patients), diarrhea, fever, and pruritus (each in two patients). Grade 2 treatment-related adverse events, experienced by at least two patients, included fatigue, decreased platelet count, and maculo-papular rash. Five patients had grade 3 drug-related AEs, the most frequent being neutropenia (in three patients), thrombocytopenia, and hypertension (each in two patients) (Table 3). One patient experienced a grade 4 decrease in lymphocyte count. There were no grade 5 adverse events. Five patients had at least one dose omission, and one patient had at least one dose modification due to toxicity. Seven Serious AEs (SAEs) were reported in five patients, and in three of them were considered drug-related and consisted of grade 3 febrile neutropenia, grade 3 and grade 2 lung infection, and grade 3 upper respiratory infection.

### 3.4. Antitumor Activity

The best response to trial treatment was CR in two patients (both FL) and PR in two patients (*n* = 1 HGBCL; *n* = 1 FL). At 6 months, two patients responded (1 CR and 1 PR); at 12 months, only one patient underwent assessment, which confirmed CR. Four patients experienced a PFS event, and PFS time ranged from 1 day (no tumor assessment post baseline) to 11.2 months.

### 3.5. Transcriptomics

**Figure 1:**
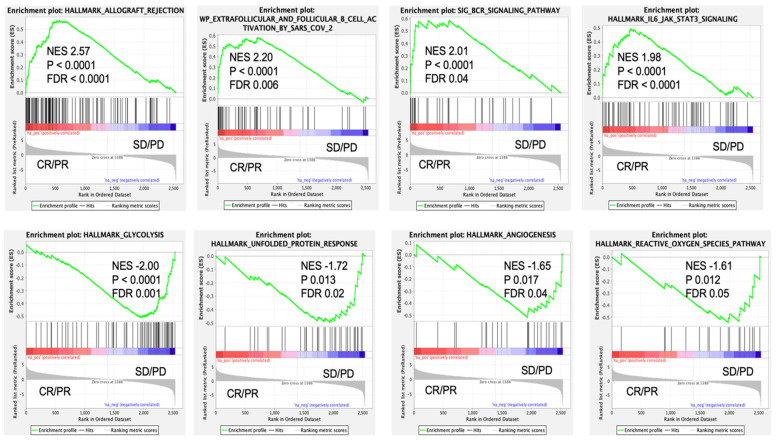
Representative GSEA plots illustrating the enrichment of transcriptional expression signatures of the genes in samples derived from responding (**upper panel**) and non-responding (**lower panel**) patients. Green line, enrichment score; bars in the middle portion of the plots show where the members of the gene set appear in the pre-ranked list of genes; positive or negative ranking metrics indicate correlation or inverse correlation with the profile, respectively. NES, normalized enrichment score. In each plot, the left panel shows genes upregulated in responders, while the right panel shows genes more highly expressed in non-responders. CR, complete response; PR, partial response; SD, stable disease; PD, progressive disease. Full data are presented in Supplementary Table S1.

Transcriptome profiling was obtained from tumor specimens for all seven patients enrolled in the study. The transcripts most highly expressed in samples from responding patients (CR/PR) showed a statistically significant enrichment for genes encoding proteins involved in BCR signaling, including *CD22*, *SYK*, *BTK*, *PIK3CD*, *CD79A*, and *ITGA4* (Figure 1, Supplementary Table S1). Conversely, genes involved in glycolysis, unfolded protein response, angiogenesis, and reactive oxygen species were enriched in samples derived from non-responders (Figure 1, Supplementary Table S1). These included *MGST1*, *VEGFA*, and *XBP1*.

## 4. Discussion

Several PI3K inhibitors have been developed for the treatment of NHL patients, including copanlisib, a pan-PI3K inhibitor with dominant activity against PI3Kα and PI3Kδ, initially approved for patients with R/R FL and MZL [10,23]. However, based on the results of the phase III CHRONOS-4 study that failed to report an improvement in PFS of copanlisib in combination with chemotherapy versus chemotherapy alone [9], the copanlisib-producing company voluntarily withdrew the FDA indication of the drug in FL, which was followed by the EMA’s removal of its orphan drug indication for R/R MZL.

BCL-2 inhibitor venetoclax has proven successful in CLL [24] and myeloid malignancies [25]. Several studies have recently provided evidence that the PI3K and Bcl-xL pathways control cell death in a synergistic manner. Modulation of BCL-2 expression is an important strategy to optimize the efficacy of therapeutic agents targeting the PI3K pathway and vice versa by potentiating apoptosis. This has been tested in solid tumors [26,27] and acute myeloid leukemia [28,29], as well as in lymphomas [12,14,30]. In particular, the combination of copanlisib and venetoclax has shown synergistic activity in various lymphoma models [12,13,14]. Both compounds have been clinically tested in combinations with other drugs in patients with different lymphoma subtypes, but no clinical trial results evaluating this combination are currently available [12].

A recent example of a small clinical trial evaluating the combination of venetoclax with BTK inhibitor ibrutinib in patients with relapsed/refractory MCL provided additional evidence that the combination of small molecules with known single-agent activity may be feasible and result in high rates of remission in patients with relapsed or refractory disease [31].

Here, we report the results of a phase I combination trial of venetoclax and copanlisib in patients with different R/R NHL subtypes. Unfortunately, the study was prematurely closed due to a pharmaceutical company’s decision.

The two drugs could not be combined at DL 1 due to AEs, but also, DL -1 of the amended protocol would likely not be tolerable, despite the absence of DLTs during cycle 1, due to adverse events arising after cycle 1 and requiring dose interruptions.

Therefore, it remains unknown whether lower doses and alternative schedules (not tested due to the trial’s closure) would have resulted in a more tolerable and feasible combination. Regarding efficacy, responses were observed in patients with aggressive lymphoma, providing some evidence of the combination’s activity. Given the small number of patients, it is not possible to draw any definite conclusions about the safety or efficacy of this combination in patients with lymphoma who have failed standard therapies.

Transcriptomic profiling of pretreatment lymphoma biopsies identified signatures associated with treatment response. In particular, tumors from responding patients showed higher expression of genes involved in B-cell receptor signaling, including CD22, SYK, BTK, PIK3CD, CD79A, and ITGA4. This observation is biologically plausible, as BCR signaling represents one of the major upstream inputs activating the PI3K pathway in mature B-cell malignancies. Engagement of the BCR complex via CD79A/CD79B and downstream kinases such as SYK and BTK promotes PI3Kδ activation and PIP3 generation, ultimately supporting AKT-dependent survival, proliferation, and metabolic adaptation [32]. Therefore, lymphomas with a more pronounced baseline BCR/PI3K transcriptional program may retain a greater dependency on this signaling axis and may be more vulnerable to pharmacologic PI3K inhibition. This mechanism may also provide a rationale for combining copanlisib with venetoclax, in line with preclinical findings [12,13]. Inhibition of PI3K signaling can weaken pro-survival signals downstream of the BCR and may lower the apoptotic threshold of lymphoma cells, thereby increasing their dependence on anti-apoptotic BCL2-family proteins. In this context, concomitant BCL2 inhibition with venetoclax may facilitate mitochondrial apoptosis in tumors in which BCR/PI3K signaling is an important survival pathway. Thus, the enrichment of BCR-signaling genes among responders supports the hypothesis that the activity observed in this study may have been driven, at least in part, by the interruption of a BCR–PI3K survival program coupled with direct targeting of BCL2-mediated apoptotic resistance.

Conversely, samples from non-responding patients were enriched for pathways related to glycolysis, unfolded protein response, angiogenesis, and reactive oxygen species. These programs may reflect alternative survival states less dependent on canonical BCR/PI3K signaling or more capable of tolerating PI3K pathway inhibition through metabolic, stress-response, or microenvironment-associated adaptive mechanisms [3,33,34,35]. Although the very small sample size and the use of archival baseline material preclude definitive biomarker conclusions, these findings suggest that BCR/PI3K pathway activation could be explored as a candidate marker of sensitivity to PI3K/BCL2 inhibitor combinations. This interpretation is also consistent with previous observations in patients treated with copanlisib, in whom higher expression of PI3K/BCR-related genes was reported in FL and other indolent lymphomas [10].

Thus, our exploratory transcriptomic analyses, based on a limited number of samples and requiring future validation in larger series, suggest that baseline activation of BCR/PI3K-related pathways may be associated with response to combination treatment, supporting the biological rationale for dual PI3K and BCL2 inhibition. The clinical development of PI3K inhibitor-based combinations has been slowed by safety concerns associated with various PI3K inhibitors, including infectious, immune-mediated, and hematologic toxicities [36,37]. Several PI3K inhibitors have been subject to regulatory restrictions or indication withdrawals due to concerns about their overall benefit-to-risk profile. However, newer generations of PI3K inhibitors such as linperlisib and roginolisib are advancing in clinical evaluation as single agents or in combination with venetoclax [38,39]. The strong preclinical synergism observed with combined PI3K and BCL2 inhibition [12,14,30] suggests that this therapeutic strategy may still warrant further investigation, potentially through alternative dosing schedules, more selective agents, or biomarker-driven patient selection approaches aimed at improving the therapeutic index.

## 5. Conclusions

In conclusion, the very limited number of patients enrolled in this study, together with the inability to define an RP2D due to the trial’s early premature termination, did not allow for any definitive conclusions regarding the safety or efficacy of this combination. The two drugs could not be combined at their respective single-agent doses, and dose reductions were necessary. Although clinical responses were observed in heavily pretreated patients, including some with aggressive R/R lymphomas, these findings should be interpreted with caution due to the small sample size. Therefore, no firm conclusions regarding the regimen’s activity can be drawn, and further investigation in larger studies is needed. In addition, transcriptomic profiling before treatment should be further explored, as it may identify patients with lymphoma who would respond well to combination regimens containing PI3K and BCL2 inhibitors.

## Figures and Tables

**Table 1:** Baseline patient characteristics.

Clinical Characteristic	N (Total = 7)	%
Age at registration (years)	Median 69	Range 53–76
Sex		
Female	4	57%
Male	3	42.9%
WHO performance status		
0	2	28.6%
1	5	71.4%
Type of lymphoma		
DLBCL, NOS	2	28.6%
FL	4	57.1%
HGBCL	1	14.3%
Number of previous systemic therapies		
2	1	14.3%
3	3	42.8%
5	1	14.3%
6	2	28.6%
Previous Radiotherapy		
Yes	4	57.1%
No	3	42.9%

**Table 2:** Dose levels and dose-limiting toxicities.

DL	No. of pts Treated/No. of pts Evaluable for DLT/No. of pts with DLT	Type of DLT
DL1: copanlisib 60 mg on days 1, 8, and 15 and venetoclax 600 mg OD continuously starting on day 2 of cycle 1; cycles of 28 days for up to 12 cycles	2/2/2	-Grade 3 febrile neutropenia -Grade 3 maculo-papular rash and Grade 3 upper respiratory infection
DL -1: copanlisib 45 mg on days 1, 8, and 15 and venetoclax 400 mg OD continuously starting on day 2 of cycle 1; cycles of 28 days for up to 12 cycles	5/3/0	NA

**Table 3:** Grade ≥3 AE at least possibly related to the study treatment.

Event	Grade 3 AEs No. of pts (%)
Neutrophil count decreased	3 (42.9)
Platelet count decreased	2 (28.6)
Hypertension	2 (28.6)
Febrile neutropenia	1 (14.3)
Mucositis oral	1 (14.3)
Lung infection	1 (14.3)
Upper respiratory infection	1 (14.3)
White blood cells decreased	1 (14.3)
Maculo-papular rash	1 (14.3)

## Data Availability

The data presented in this study are available upon request from the corresponding author.

## Supplementary Materials

The following supporting information can be downloaded at: https://www.mdpi.com/article/10.3390/cancers18111764/s1, Table S1: Transcriptome analysis of samples derived from patients enrolled in the phase 1 study.
